# Supplementary material for: Neuro-glial lipid imbalance in a Drosophila model of amyotrophic lateral sclerosis 8
Source: Acta Neuropathol Commun. 2026 Jul 27;14:189. doi: 10.1186/s40478-026-02391-y (PMC13587292; doi:10.1186/s40478-026-02391-y)
Supplement: Supplementary file 1 [file 40478_2026_2391_MOESM1_ESM.pdf]

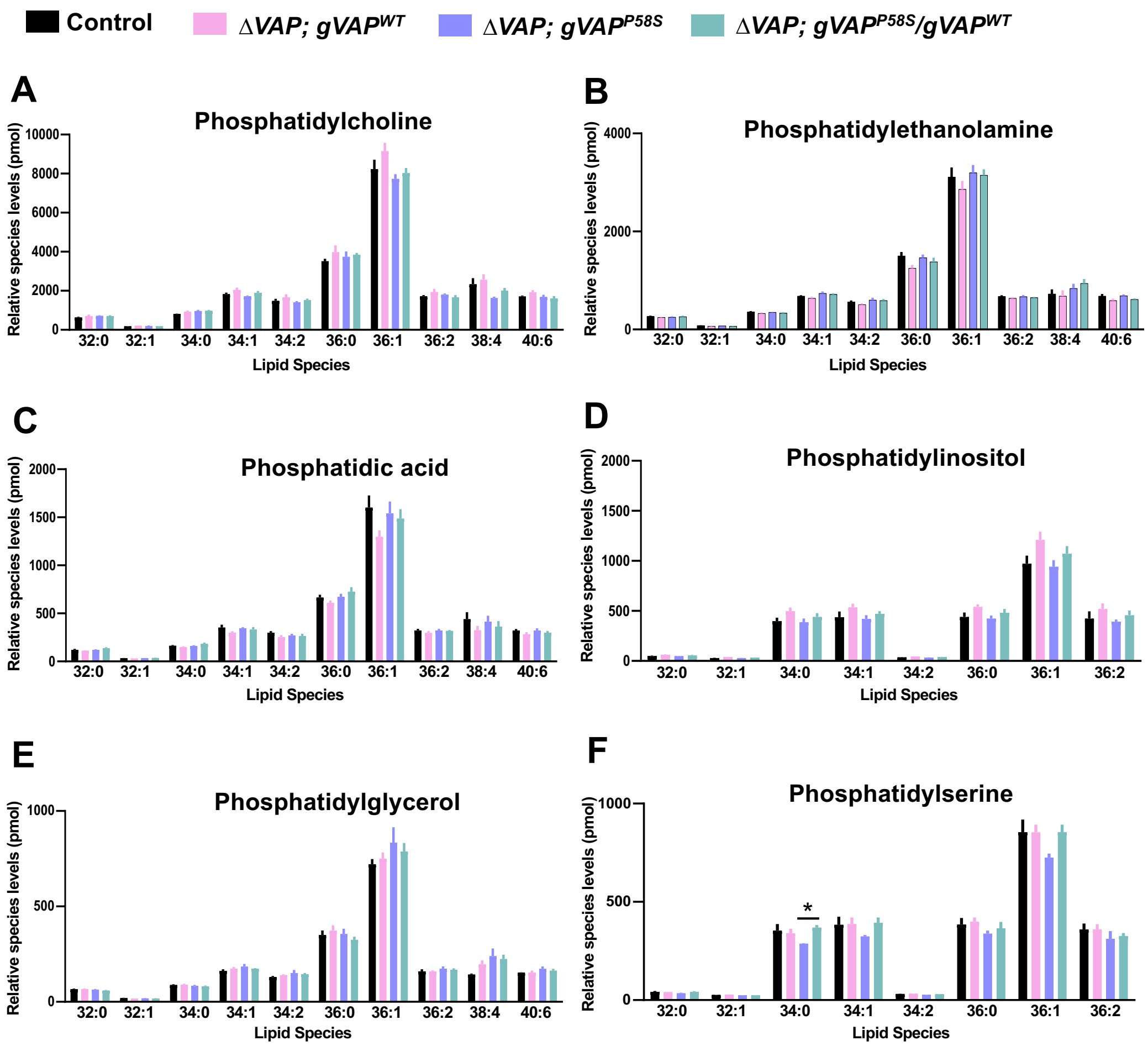

Suppl. Figure 1. LC-MS lipid quantitation for phospholipids in 15 day old *Drosophila* male brain. No significant change is detected in the phospholipid species of Δ*VAP; gVAP^{P58S}* compared to controls for (A) Phosphatidylcholine (PC), (B) Phosphatidylethanolamine (PE), (C) Phosphatidic acid (PA), (D) Phosphatidylinositol (PI), (E) Phosphatidylglycerol (PG). However, Phosphatidylserine(PS) C34:0 is significantly reduced in *ALS8* model which are rescued by single copy of *VAP* (F). Multiple unpaired t-test is performed for mean comparison between genotypes with FDR approach of Benjamin and Hochberg for multiple comparison correction with p values reported as *(<0.05), **(<0.01) and ***(<0.001). N=5 biological replicates, n=2 adult brains for each genotype.

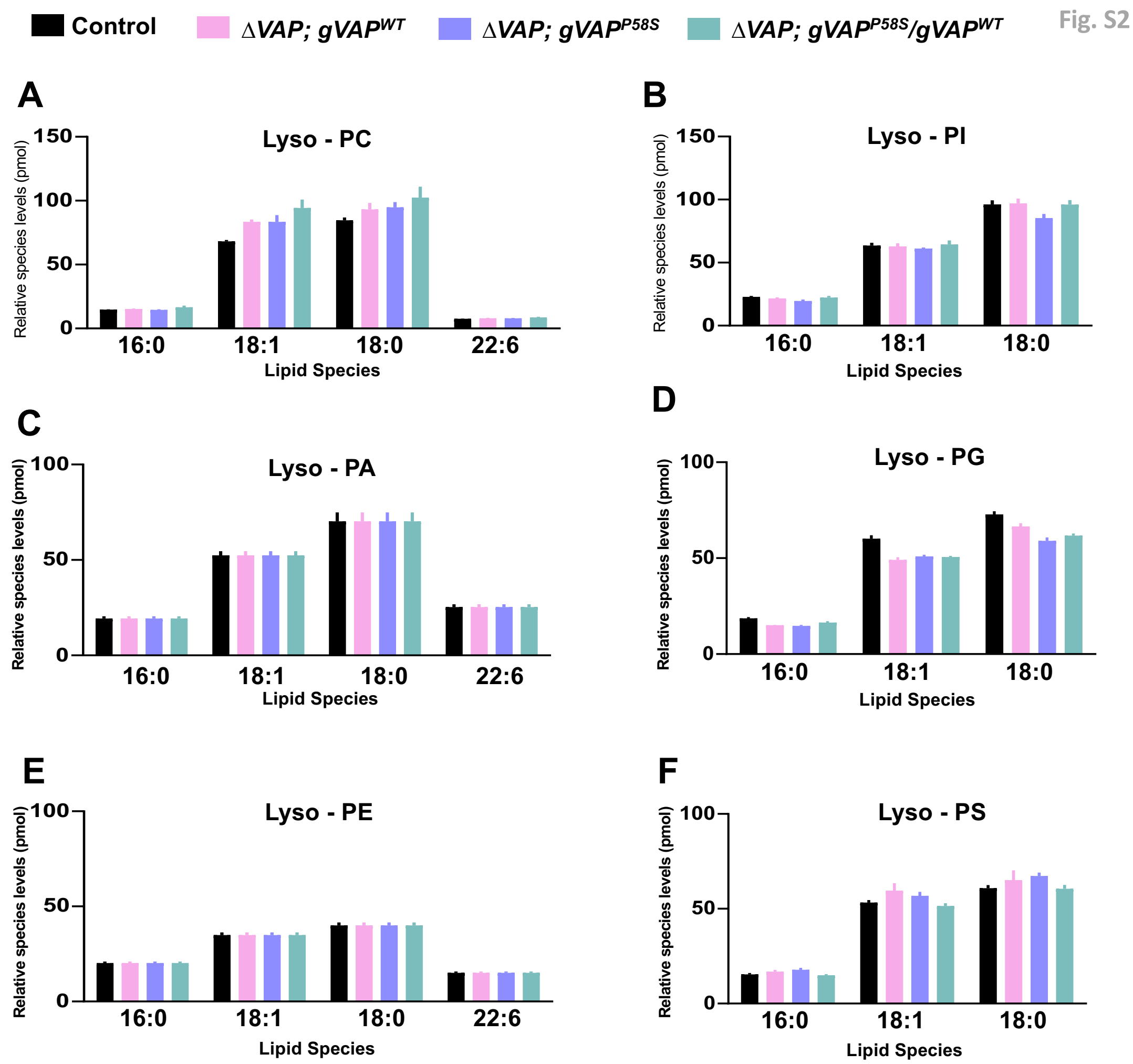

Suppl. Figure 2. LC-MS lipid quantitation for lyso-phospholipids in the 15 day old *Drosophila* male brain. No significant change is detected in lyso-phospholipid levels of *ΔVAP; gVAP^{P58S}* compared to controls for (A) Lyso-Phosphatidylcholine (Lyso-PC), (B) Lyso-Phophatidylinositol (Lyso-PI)**,** (C) Lyso-Phosphatidic acid (Lyso-PA), (D) Lyso-Phosphatidylglycerol (Lyso-PG), (E) Lyso-Phosphatidylethanolamine (Lyso-PE) and (F) Lyso-Phosphatidylserine (Lyso-PS). Multiple unpaired t-test is used as statistical test for mean comparison between genotypes with FDR approach of Benjamin and Hochberg for multiple comparison correction with p values reported as *(<0.05), **(<0.01) and ***(<0.001). N=5 biological replicates, n=2 adult brains for each genotype.

Fig. S3

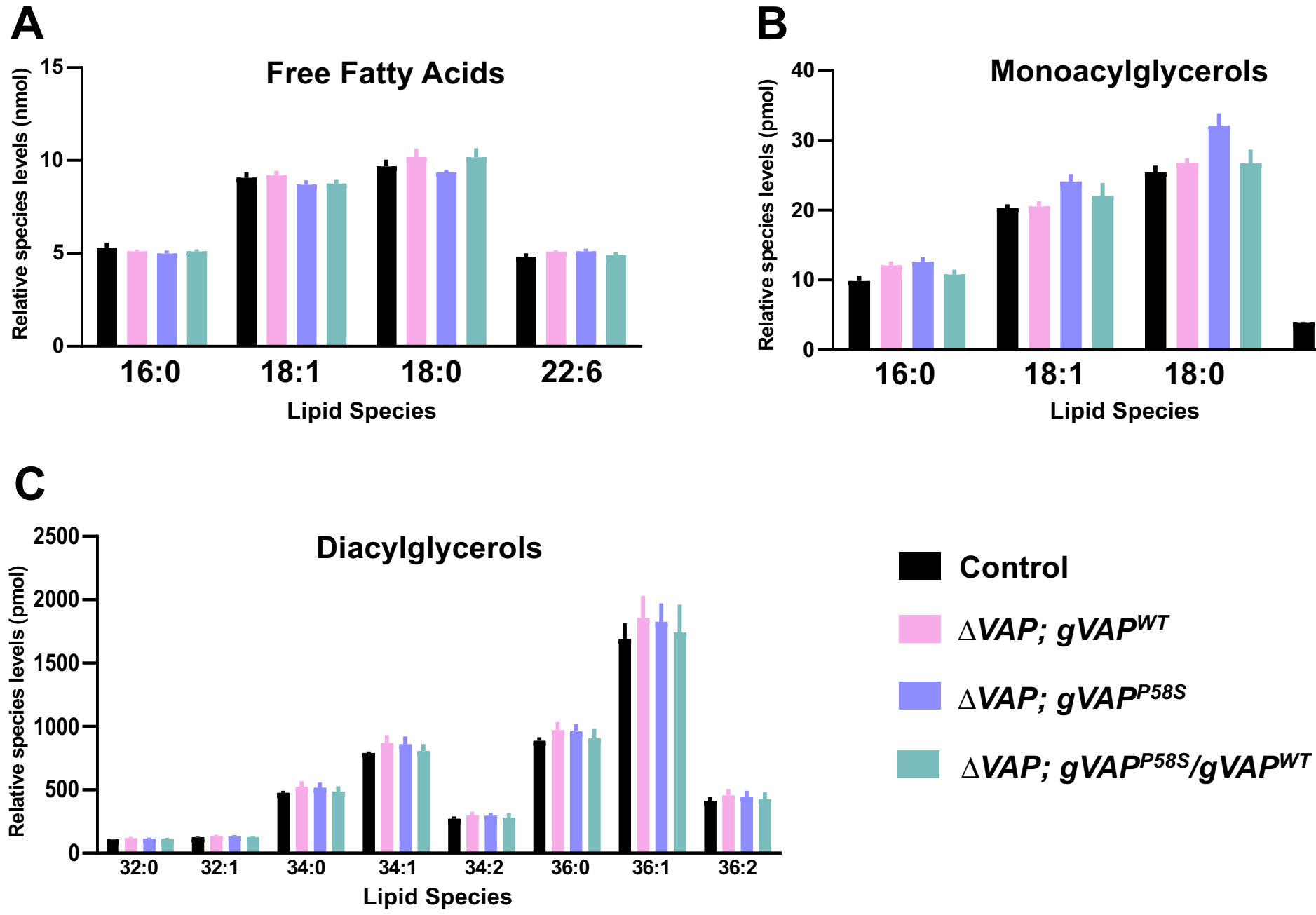

Suppl. Figure 3. LC-MS lipid quantitation in the 15 day old *Drosophila* male brain. Relative lipid/species are plotted in the graph as mean±SEM for (A) Free Fatty Acids (FFA), (B) Monoacylglycerols (MAGs) and (C) Diacylglycerols (DAGs). Lipid levels remain unchanged for the mentioned speices in the brain of Δ*VAP; gVAP*^{P58S} male flies at day 15 compared to controls. Multiple unpaired t-test is used as statistical test for mean comparison between genotypes with FDR approach of Benjamin and Hochberg for multiple comparison correction. N=5 biological replicates, n=2 adult brains for each genotype.

Fig. S4

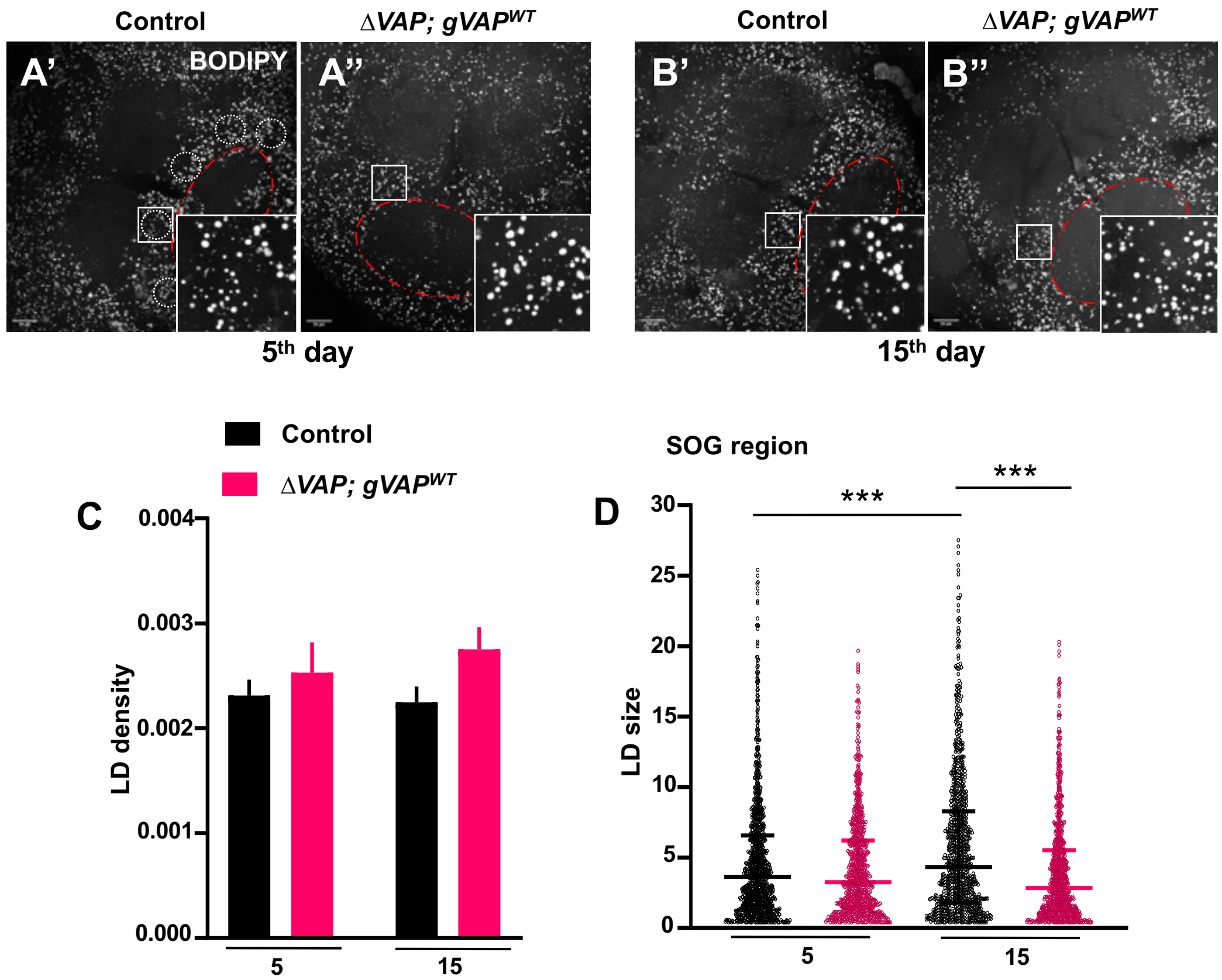

Suppl. Figure 4. No change is detected in LD density of Δ*VAP; gVAP*^{WT} *Drosophila* adult brains.
(A-B) Representative MIPs of BODIPY-stained LDs in the male adult brain's central body with a zoomed inset shown in the lower right corner. Scale bar is shown as 20 µm. (A) LD distribution is shown at 5th day for wild-type control (A') and Δ*VAP; gVAP*^{WT} (A''). Similarly, (B) represents the distribution for 15-day-old male flies. SOG region is selected for the analysis of LDs depicted by red outline. ROIs are represented around SOG region in control (A) used for LD quantitation.
(C) Quantitation for LD density (number of droplets per µm^3) plotted as mean±SEM values on Y axis against age (days) on X axis. Two way ANOVA was performed for statistical test between genotypes followed by Tukey's test for multiple comparison correction with p values reported as *(<0.05), **(<0.01) and ***(<0.001).
(D) Scattered dot plot for LD size (volume, µm^3) is plotted with size on the Y axis, including median with interquartile range, against age (days) on the X axis. Kruskal-Wallis test was used for mean rank comparison between genotypes, followed by Dunn's test for multiple comparison correction with p values reported as *(<0.05), **(<0.01) and ***(<0.001). N=5 adult brains per genotype, n= 5 ROIs analyzed for each brain.

Fig. S3

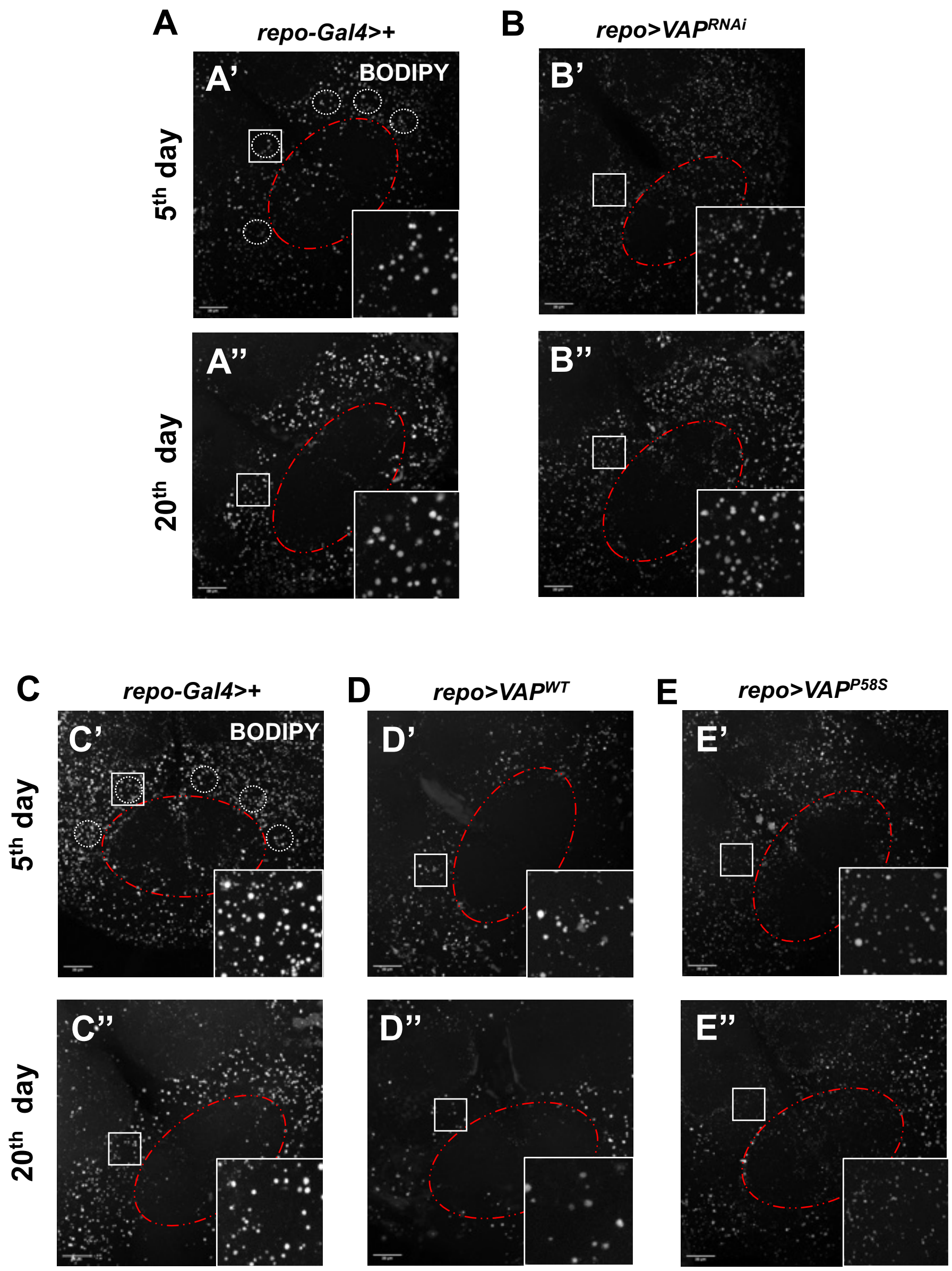

Suppl. Figure 5. ***VAP*** **is a negative regulator for LD distribution in the glial cells of** ***Drosophila*****.**
(A-B) shows MIPs (20% Brightness) of BODIPY-stained adult brain SOG region, with zoomed inset on the lower right corner. Scale bar = 20µm. The SOG region is selected for LD analysis, as depicted by a red outline. ROIs are represented around the SOG region in the control (A') used for LD quantitation. N=5-6 adult brains, n=5 ROIs for each brain. **(C-E)** are representative images (MIP, 20% Brightness) of BODIPY-stained LDs in the adult *Drosophila* brain (SOG region) of 5 and 20-day-old male flies following glial *VAP* modulation. Zoomed insets are shown in the lower right corner of each image. Scale bar is represented as 20µm. Five ROIs are drawn around SOG region (outlined by red color) in control (C') used for LD analysis. N=5 adult brains, n=5 ROIs for each brain.

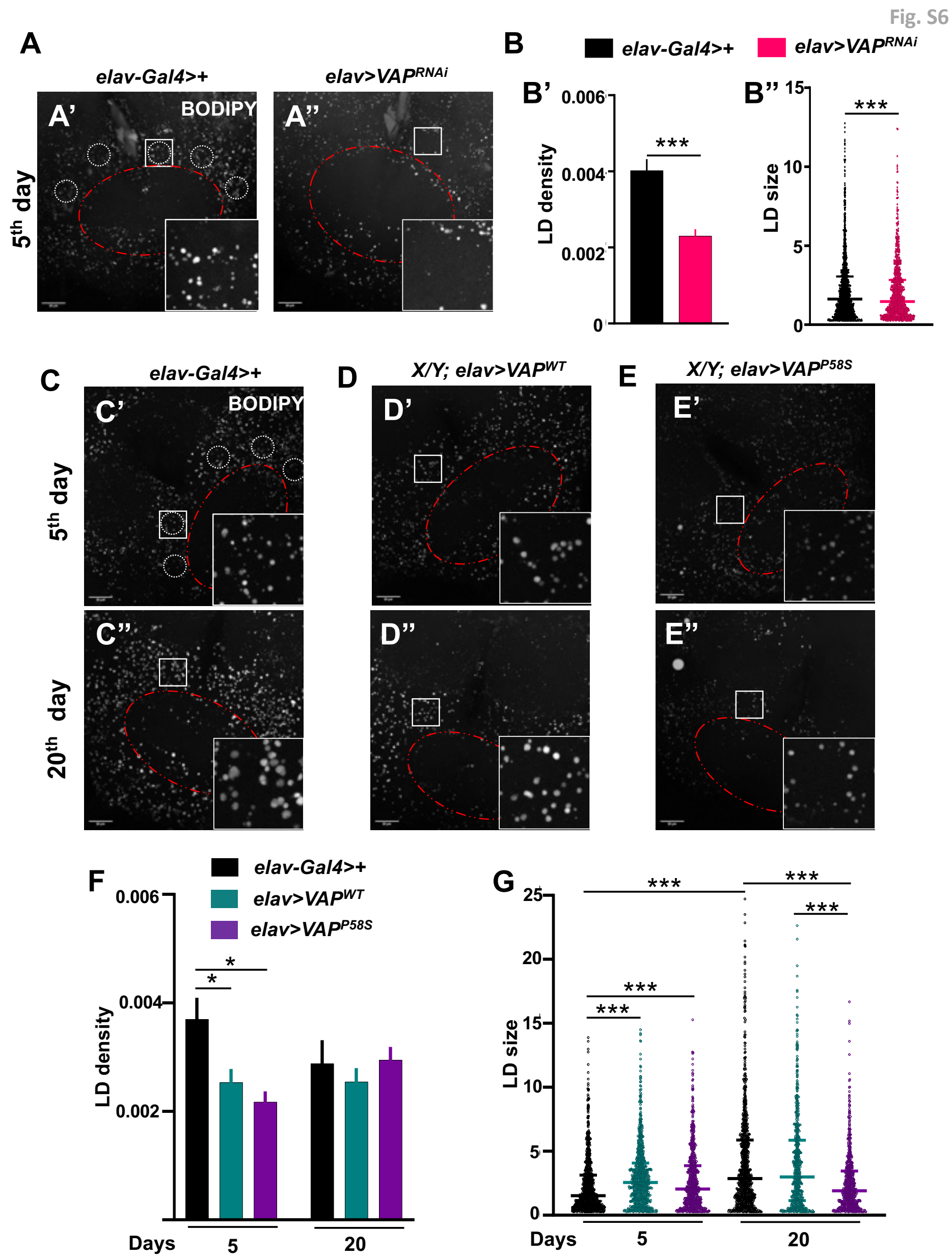

Suppl Figure 6. Effect of neuronal *VAP* modulation on the adult brain LD homeostasis.
(A) shows MIPs (20% Brightness) of BODIPY stained LDs in the SOG region of the 5 day old *Drosophila* adult brain with zoomed inset on the lower right corner. Scale bar = 20µm. (A') shows LD distribution in the adult brain for *elav-Gal4* control whereas (A') shows reduced LD distribution on *VAP* KD in the neuronal cells of the male flies. Five ROIs are drawn around SOG region (outlined by red color) in control (A') used for LD analysis.
(B) Quantitation of LD distribution is plotted using LD density (number of LDs per µm^3) and LD size (volume in µm^3) as parameters. (B') Mean±SEM value is plotted on Y axis as LD density. Unpaired t-test is used as statistical test for mean comparison between genotypes followed by Welch's test with p values reported as *(<0.05), **(<0.01) and ***(<0.001). (B'') Scattered dot plot is shown for LD size for control and *VAP* knockdown in neurons along with median and interquartile range value. Non-parametric t-test for LD size is used for mean rank comparison

followed by Mann Whitney test, p-values are mentioned using APA style. N=5-6 adult brains, n=5 ROIs for each brain.

(C-E) are representative images (MIP, 20% Brightness) of BODIPY stained LDs in the adult *Drosophila* brain (SOG region) of 5 and 20 day males flies following neuronal *VAP* modulation. Zoomed insets are shown in the lower right corner of each image. Scale bar is represented as 20µm. Five ROIs are drawn around SOG region (outlined by red color) in control (C') used for LD analysis. (C) shows images for Gal4 control of 5 day old (C') and 20 day old (C") brain samples. **(D)** represents images for LD distribution on overexpressing VAP^{WT} in the neurons for 5th day (D') and 20th day respt. (D"). **(E)** VAP^{P58S} OE in the neurons of *Drosophila* leads to changes in LD size on day 5 (E') as well as day 20 (E") **(F)** Quantitation of LD density (number of droplets per μm^3) is plotted as mean±SEM value in the graph. Two way ANOVA is used for mean comparison between genotypes of same day followed by Tukey's test for multiple comparison correction with p values reported as *(<0.05), **(<0.01) and ***(<0.001).

(G) Quantitation of LD size (volume in μm^3) is plotted as scattered dot plot along with median and interquartile range. Kruskal Wallis test is used for mean rank comparison between genotypes followed by Dunn's test for multiple comparison correction with p values reported as *(<0.05), **(<0.01) and ***(<0.001). N=5-6 adult brains, n=5 ROIs for each brain.

Fig. S7

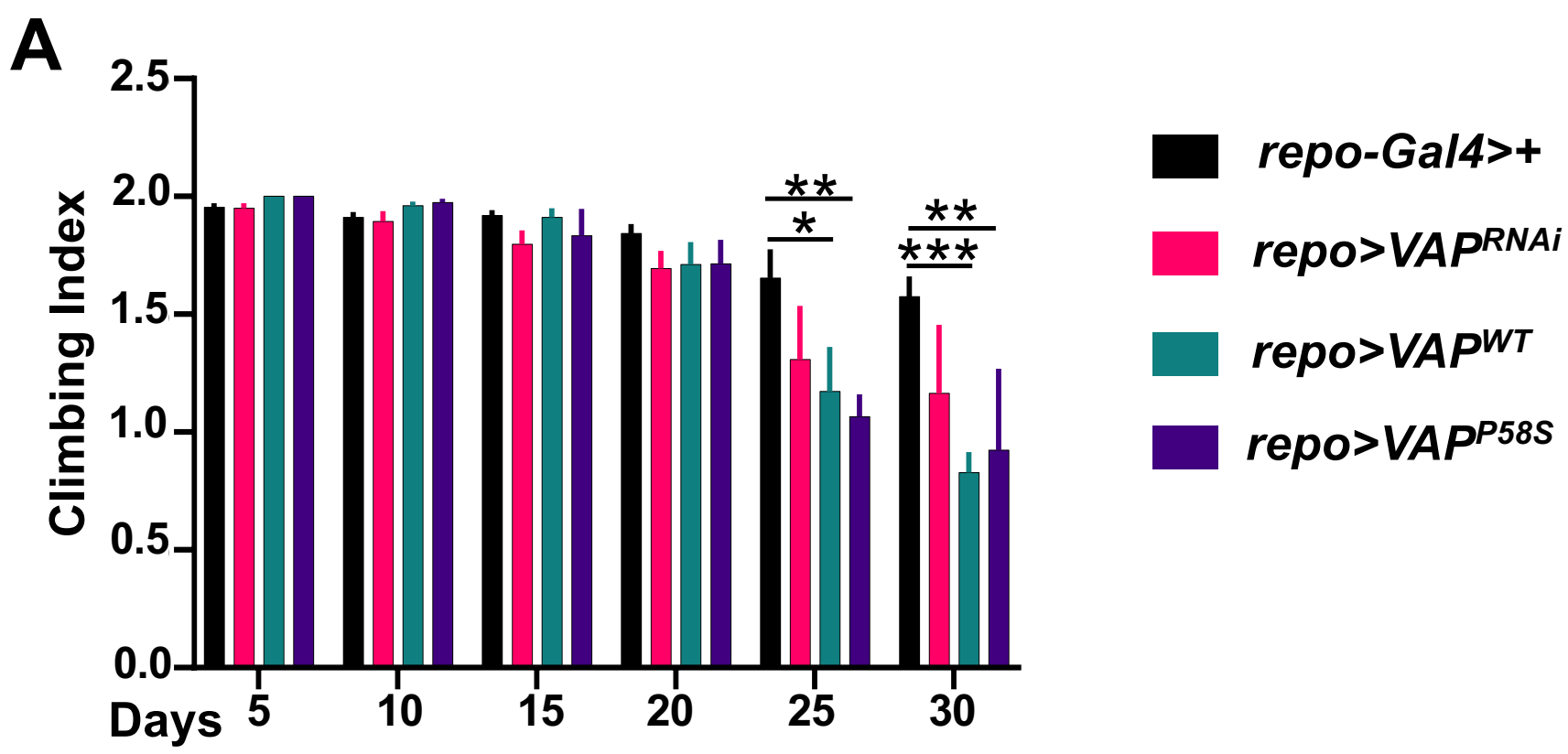

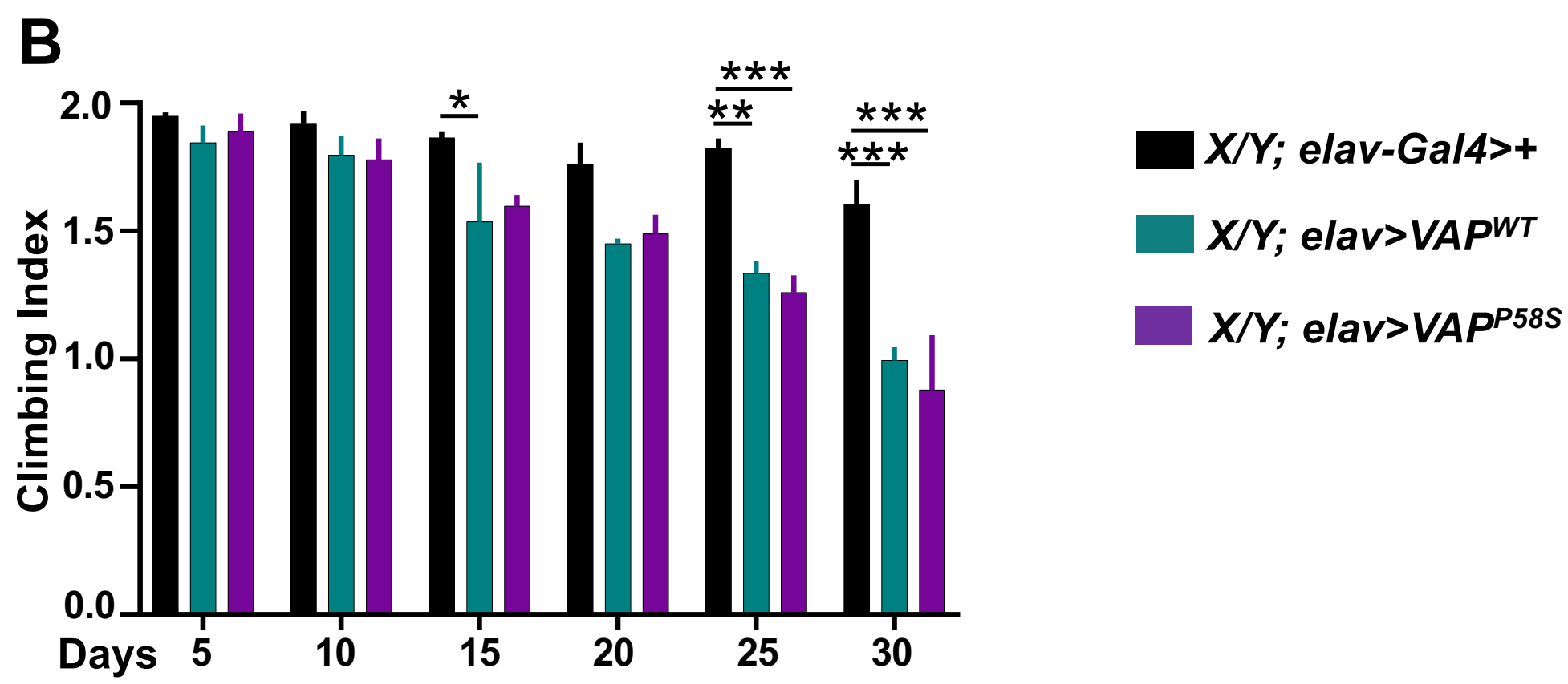

Suppl. Figure 7. Effect of *VAP* modulation on the motor function of the flies.
(A) Climbing Index is plotted for the motor function on modulating *VAP* in the glia of *Drosophila.* Removal of *VAP* in the glial cells does not appear to affect climbing activity of the flies, but more VAP, either wildtype or mutant (VAP^{P58S}), leads to significant motor impairment day-25 onwards.
(B) Climbing Index is plotted for the motor function on modulating *VAP* in the neurons of *Drosophila.* VAP^{WT} OE in the neuronal cells leads to motor defects on day 15 onwards whereas VAP^{P58S} OE leads to significant motor dysfunction on 25th day onwards.

Two-way ANOVA was performed for mean comparisons between genotypes on the same day, followed by Tukey's test for multiple comparison correction with p values reported as *(<0.05), **(<0.01) and ***(<0.001). N=3 biological replicates, n~30 male flies for each genotype.

Fig. S8

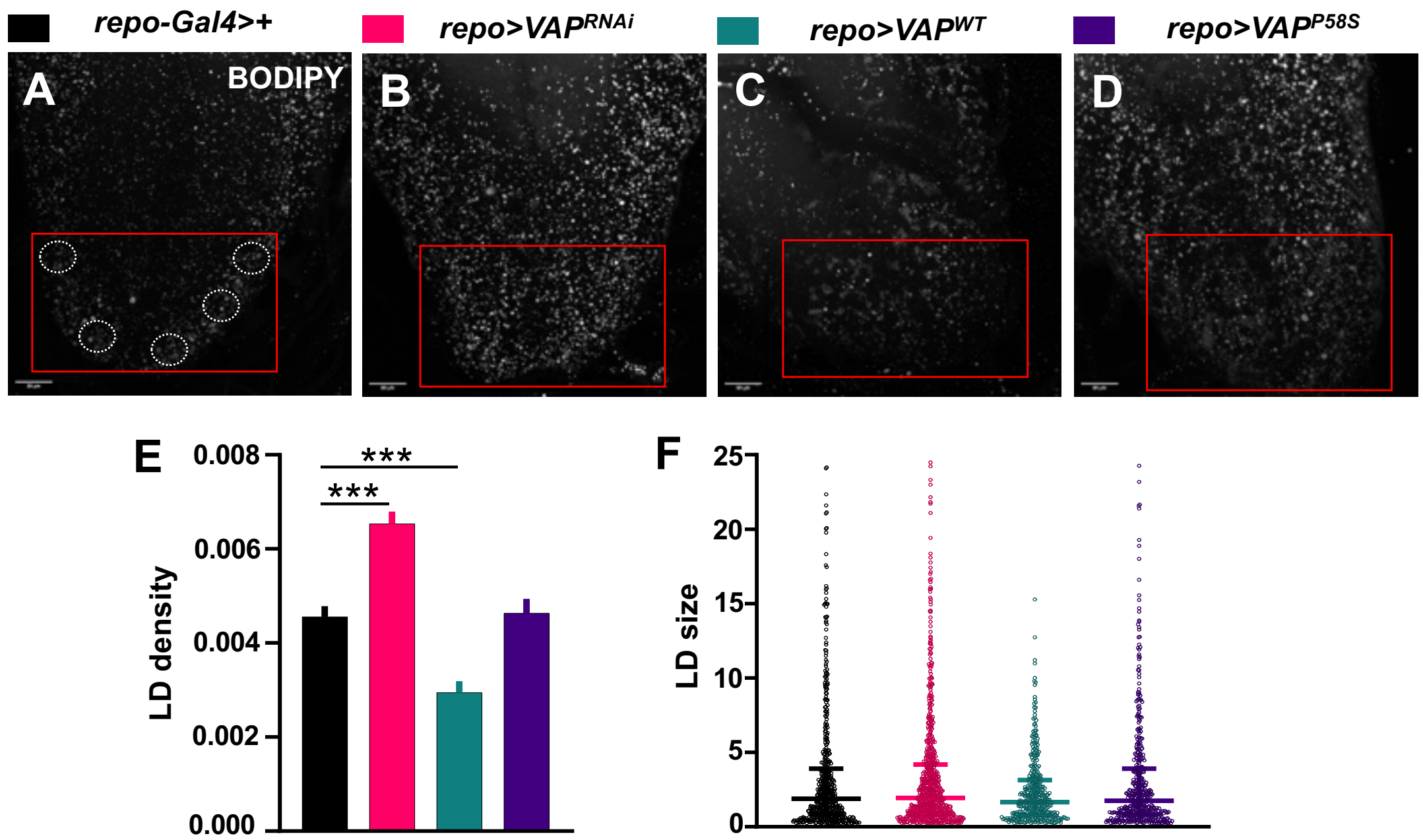

Suppl. Figure 8. Glial *VAP* modulation affects LD homeostasis in the larval brain.

(A-D) represent MIPs (20% Brightness) of the ventral nerve cord of the late 3rd instar male larval brain stained with BODIPY. Scale bar = 20µm. Five ROIs are drawn at the tip of the VNC, outlined in red in the control (A), and used for LD analysis. *VAP* KD in glia shows an increase in LD number compared to control. Overexpression of VAP^{WT} leads to a decrease in LD density, whereas VAP^{P58S} OE shows no change compared to the control.

(E) Quantitation of LD density (number of droplets per µm^3) is plotted as Mean±SEM values in the graph. One-way ANOVA is used for mean comparison between genotypes, followed by Tukey's test for multiple comparison correction, and p values are reported as *(<0.05), **(<0.01) and ***(<0.001).

(F) LD size (volume in µm^3) is plotted in the form of a scattered dot plot representing LDs present in ROIs of each genotype. The Kruskal-Wallis test was performed for mean rank comparison between genotypes, followed by Dunn's test for multiple comparison correction. N=4-5 larval brains per genotype, n= 5 ROIs for each brain sample.
